# Nurturing Sustainability and Health: Exploring the Role of Short Supply Chains in the Evolution of Food Systems—The Case of Poland

**DOI:** 10.3390/foods12224171

**Published:** 2023-11-19

**Authors:** Nina Drejerska, Wioleta Sobczak-Malitka

**Affiliations:** Institute of Economics and Finance, Warsaw University of Life Sciences, 02-787 Warsaw, Poland; nina_drejerska@sggw.edu.pl

**Keywords:** food systems, short supply chain, healthy eating, food and health, food quality, consumer health, sustainable nutrition, local production, socio-ecological systems, food security and sovereignty

## Abstract

Over the last few decades, short food supply chains and local food markets, where farmers either sell their products directly to consumers or use a limited number of intermediaries, have developed worldwide in rural and urban areas. They complement conventional, often globalized, long food chains where small farmers have little bargaining power, and consumers cannot link the food they buy to a known agricultural producer or geographical area where the food is produced. The advantage of direct sales is that producers can obtain a higher price while consumers have easier access to fresh and seasonal food products. The main aim of the paper is to identify and characterize the spatial concentration of local food systems in Poland and their importance in sustainable development and food policy for healthy eating. As part of this study, an analysis of the statistical data of the Central Statistical Office for 2021 was carried out. Data obtained from the Chief Veterinary Inspectorate as of mid-2021 were analyzed to discuss the topic in detail. Descriptive methods and comparative analyses were used to understand regional differences. Absolute and proportional values were used for the research to enable better comparisons between regions, using the traditional method used in spatial structure studies, i.e., the distribution index (number of entities per 1000 inhabitants). The analysis identified spatial differences and possible implications for food policy and regional development. In addition, data on the number of marketplaces in Polish regions in 2022 were used. The study results indicated that short supply chains in the Polish food system contribute to increasing the availability of healthy local products, which may improve consumer health. However, despite these benefits, the results revealed challenges such as the limited production scale of local suppliers and the need to adapt to changing market conditions.

## 1. Introduction

### The Importance of Short Supply Chains in Sustainable Development

Terms such as “local food” and “local food system” are often used interchangeably to refer to food produced close to its place of consumption. The main characteristics that consumers attribute to local food are sustainable production and distribution practices, while the use of fertilizers and plant protection products is reduced. Sometimes, consumers also extend sustainable production and distribution to include fair farm work practices and animal welfare. Local food can also refer to a producer: the breeder’s personality and ethics, the farm’s attractiveness, and the surrounding landscape. It corresponds well to a broader model of food production in Poland, wherein agricultural entrepreneurs are emotionally devoted to their resources [1]. The essence of local food products is also that they reach consumers via short supply chains. Short supply chains facilitate the link between the consumer and the food producer by providing more precise information on the origin of food products. This enables consumers to identify with the production site and the people involved.

There was no standard definition of short food supply chains at the EU level for a long time. However, they are commonly understood to involve a minimum number of intermediaries (or even direct sales from the producer). Currently, now the EU has recognized short supply chains as a matter that should be supported in rural areas, they are covered by the definition in Regulation (EU) No 1305/2013 of the European Parliament and of the Council of 17 December 2013 on support for rural development by the European Agricultural Fund for Rural Development (EAFRD) [2], which entered into force with the reformed Common Agricultural Policy for 2014–2020. According to this regulation, a short supply chain means a supply chain that includes a limited number of cooperating economic actors, brings local economic development, and is characterized by close geographic and social links between producers, processors, and consumers [2]. This definition is supplemented by Commission Delegated Regulation (EU) No 807/2014 [3], which indicates that the support for the establishment of short supply chains referred to in Art. 35 sec. 2 lit. (d) Regulation (EU) No 1305/2013 covers only supply chains where no more than one intermediary is involved between the farmer and the consumer.

However, defining the local food system is more complex. Regulation (EU) No 807/2014 only provides the definition of local markets eligible for EAFRD support, as defined in the Member States’ rural development programs [3]. The local food system assumes food production, processing, and selling within a defined geographical area. Examples of local food systems are agricultural markets, farm sales, boxed vegetable delivery systems, community-supported agriculture, and public procurement systems that source food within a specific geographic radius. Food listed in local food systems is usually traceable to a particular place of provenance and has distinctive features. These are often unprocessed or slightly processed commodities. So far, there is no legally agreed definition of local food or the geographical level of this process. Locality is often referenced and understood in relation to larger geographic scales, such as regional, national, or global ones.

As there is no universal definition of local food, distinguishing the types of local markets facilitates their analysis and evaluation. Two basic types of local food markets are listed in the state of the art. The first covers markets where transactions are carried out directly between farmers and consumers (food products go straight from the producer to the consumer). The second refers to markets where farmers sell food directly to restaurants, retail stores, and institutions such as government units, hospitals, and schools (direct sales to retail/catering) [4].

The main aim of the work is to identify and characterize the spatial concentration of local food systems in Poland and their importance in sustainable development and food policy for healthy eating. The paper has the potential to bring new light to current gaps in understanding the role of short supply chains in the context of the food system in Poland. By examining in detail the specific local and regional conditions, unique challenges, and benefits, the article can expand the current knowledge on the complex relationships between short supply chains and the evolution of the food system. Furthermore, it provides practical implications for policymakers and practitioners involved in developing sustainable food strategies. By analyzing the economic benefits for local producers, the impact on consumer health and innovation practices in Poland, the article can serve as a guide for decision makers who seek to promote sustainability in the food sector. In summary, the scientific validity of this paper lies in its potential to advance sustainability theory, especially in the context of global challenges. Exploring how short supply chains can contribute to solving global issues such as climate change and social justice can serve as inspiration for future research and the development of new theoretical approaches. In this way, the paper not only expands existing knowledge, but also opens the door to further exploration, supporting scientific progress in the fields of sustainability and food.

## 2. Materials and Methods

**Figure 1:**
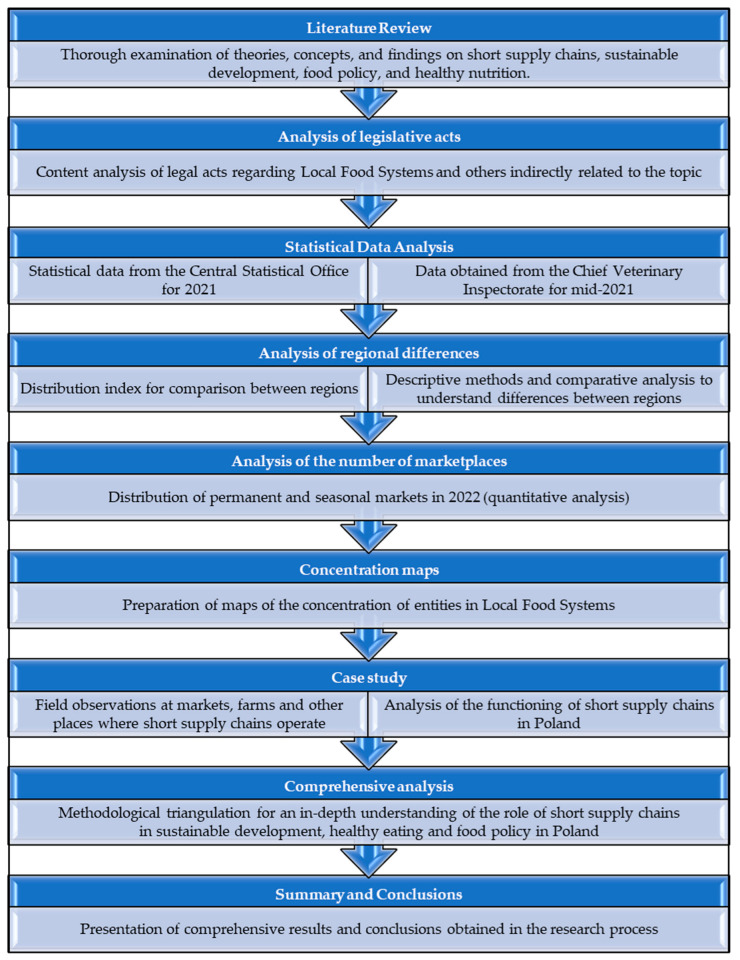
Methodological diagram. Source: Own study.

This paper presents a comprehensive research methodology for analyzing short supply chains’ role in sustainable development, food policy, and healthy eating in Poland. The sequence of procedures in this study is presented in the diagram below (Figure 1).

The research began with an in-depth literature review to identify existing theories, concepts, and lessons learned on short supply chains, sustainability, food policy, and healthy eating. By analyzing various sources, from scientific articles to reports from international institutions, we aimed to understand the key and missing links in the context of Poland. Our initial set of materials included over 100 works from publicly available databases. However, due to the detailed topic of the work, only the selected items are presented. The keywords used for searching were as follows: short supply chains, sustainability, food policy and healthy eating, development of distribution channels, changes in distribution, and from farm to fork. The content of legal acts concerning the topic of Local Food Systems and legal acts indirectly related to this subject was also analyzed. As part of this study, an analysis of the statistical data of the Central Statistical Office for 2021 was carried out. Coming from the Local Data Bank, i.e., a publicly available database, these data concerned the number of marketplaces and the level of agricultural production in Poland. Data obtained from the Chief Veterinary Inspectorate in response to an inquiry submitted to this office as of mid-2021 were analyzed to discuss the topic in detail. The material provided included the number of registered entities that sell in the form of short supply chains, divided into individual product categories and the location of these entities.

Data on the number of entities registered under the LSF were presented for Poland and its regions. Descriptive methods and comparative analyses were used to understand regional differences. The current status of short supply chains was indicated and Polish regions were compared in order to determine similarities and differences in the number of short supply chains. Absolute and proportional values were used for the research to enable better comparisons between regions, using the traditional method used in spatial structure studies, i.e., the distribution index (number of entities per 1000 inhabitants). The analysis identified spatial differences and possible implications for food policy and regional development. In addition, data on the number of marketplaces in Polish regions in 2022 were used. The quantitative analysis focused on determining the total number of markets and their distribution by region. Additionally, the areas with the largest and smallest number of permanent and seasonal markets were analyzed, indicating the number of these solutions. Concentration maps were prepared to present the spatial concentration of entities operating in Local Food Systems.

The paper includes a case study of Poland, referring to forms of short supply chains in the context of sustainable development, the availability of healthy food and support for local farmers. A case study approach is an in-depth analysis of a specific area, in this case Poland, to understand the details, context, and implications of short supply chain practices. This case study provides concrete examples of short supply chain solutions in Poland, enabling the identification of practical implications for local actions and policymakers. In the preparation of the study, the method of field observation at marketplaces, farms, and other places where short supply chains operate was also used. Observations run by the authors for the last 15 years helped to understand the practical aspects of short supply chains. To sum up, the research methodology is based on diverse approaches, enabling an in-depth analysis of the role of short supply chains in the context of sustainable development, healthy eating, and food policy in Poland. A combination of different methods (methodological triangulation) resulted in a comprehensive approach to the problem investigated.

## 3. Literature Review

### 3.1. Local Food Markets in the Context of Changes in the Food System

The analysis and evaluation of local food markets should be seen in the context of general changes in food systems. As the latest research shows, global and local transformation of the food system is necessary to ensure the provision of healthy, safe, and nutritious food sustainably and equitably [5]. Food systems are complex concepts that influence diet, human health, and other areas of society, including economic growth, natural resources and environmental resilience, and socio-cultural factors. However, food systems contribute and are vulnerable to ongoing climate and environmental changes threatening their sustainability. The relationship between food systems and the environment is complex, as environmental change is both a driving force and the result of the functioning of food systems. Environmental factors such as soil and water quality, weather, and temperature influence food production, storage, and transport. This affects the local food market—where consumers interact with the food system to buy and eat food.

The specificity and nature of the short supply chains underlying local and regional food markets make them flexible and closer to local communities [6]. The literature emphasizes that management rigidity in long, global food supply chains underlies the weakness of the food supply system and that its resilience in an era of continued uncertainty surrounding both retail supply chains and catering services will require flexible strategies [7].

Among the market forces driving the demand for local food products, the ability of local food companies to respond flexibly to changes in consumer demand for unique food and drink characteristics and varieties is essential [8,9]. The nature of the linkages between stakeholders is also an important and innovative feature of local food markets, which have the potential to facilitate connections between producers, consumers, and small food businesses, as well as public sector stakeholders (health, universities, and government agencies) [10]. As emphasized in many studies, the basis for developing and strengthening local food systems is the development of social capital [11,12]. Social benefits also include the fact that they contribute to social inclusion and improve the quality of life of local communities. Altering the agri-food chain within a focused network distinguished by robust communication and relationships enables the appropriate positioning and roles to be assigned to various participants in the chain, which can result in the creation of a balance, with the ultimate goal of reducing food loss and waste [13]. In turn, environmental benefits result from agricultural producers’ more sustainable and environmentally friendly behavior.

The role of local food systems in revitalizing rural economies is increasingly being appreciated. The positive consequences of their functioning are of interest to authorities at the local level and the European Community, especially since local food systems can be part of the broadly understood processes of sustainable rural development [14]. The purchase of local products within short food supply chains, i.e., through occasional or regular food fairs or sales at fairgrounds, is quite a common way of food purchases. This is in line with the current megatrends in the food market: health, comfort, and pleasure. In this context, it is emphasized that short food supply chains’ benefits have an economic, health, social, and environmental character [15]. In the context of the various challenges of the modern world economy, which include, in particular, population growth, climate change, and the loss of food production areas in favor of growing crops for energy purposes—the development of local food systems, meaning production, processing, and marketing in a relatively small area, is of vital importance. It significantly contributes to solving these problems and is a crucial element of sustainable development worldwide [16].

Sustainable supply chain management (SSCM) is defined as “The creation of coordinated supply chains through the voluntary integration of economic, environmental and social aspects with key inter-organizational business systems designed to efficiently and effectively manage material, information and capital flows related to the procurement, production and distribution of products or services to meet stakeholder requirements and improve the profitability, competitiveness and resilience of the organization in the short and long term” [17]. It should be noted that the circular economy pushes the boundaries of environmental sustainability by emphasizing the idea of transforming products so that there are workable relationships between ecological systems and economic growth. Therefore, the circular economy is not only about reducing the use of the environment as a sink for residues, but rather about creating self-sufficient production systems where materials are used repeatedly. The integration of the principles of the circular economy as part of sustainable supply chain management can bring clear benefits for environmental protection and thus also have a positive impact on health aspects that are significantly dependent on the state of the environment, which also indirectly affects food quality [18]. It should be noted that short supply chains represent a sustainable alternative to global chains in terms of economic, social, and environmental benefits [19].

The complex and lengthening food supply chain increases the risks associated with food quality. The sustainable development-oriented supply chain is an extension of the green supply chain, which also takes into account social criteria, including health, along with economic and ecological criteria [20], thus becoming an integral part of the planning process of modern production to improve supply chain efficiency [21]. As Migliore and co-authors [22] pointed out, short food supply chains (SFSC) are characterized by certain appropriate features of production quality assurance, which ensures consumers that the products are of good quality.

In recent years, the interest in purchasing food products through SFSC has steadily grown in Poland and throughout Europe [23]. It should be noted that more and more attention is now being paid to consumer confidence in food choices. In addition, the progressing industrialization and globalization of agri-food chains have increased consumer skepticism regarding food quality and safety [24]. The use of solutions such as short delivery channels is in line with the current critical and ethical consumerism, which is strongly related to both the environmental and health impact of food consumption and its effect on health [25].

### 3.2. Benefits and Challenges for the Development of Short Supply Chains

When analyzing benefits of short food supply chains, it should be emphasized that cooperation within short sales channels positively affects producers and consumers. For farmers, selling agricultural products directly or through short supply chains allows them to maintain a higher share of the final selling price. It can also be a significant source of income, enabling investment activities related to the extension or modernization of the farm. These channels also benefit consumers who receive fresh products from an identifiable and known producer. In addition, such local markets enable people with low incomes to buy healthy food at an affordable price, and, particularly important nowadays, they allow for building long-term partnerships between food producers and consumers.

It should also be emphasized that short supply chains are a direction for developing food distribution, which supports sustainable food systems and increases their resistance to shocks occurring in global markets. Direct food distribution models are part of sustainable development by considering all its dimensions, i.e., social, economic, and environmental benefits. Short supply chains provide benefits for farmers and consumers, supporting the development of a more sustainable food system that considers today’s social and environmental challenges. They can also improve the competitiveness and overall stability of the agri-food system in individual countries, regionally and globally [26].

**Figure 2:**
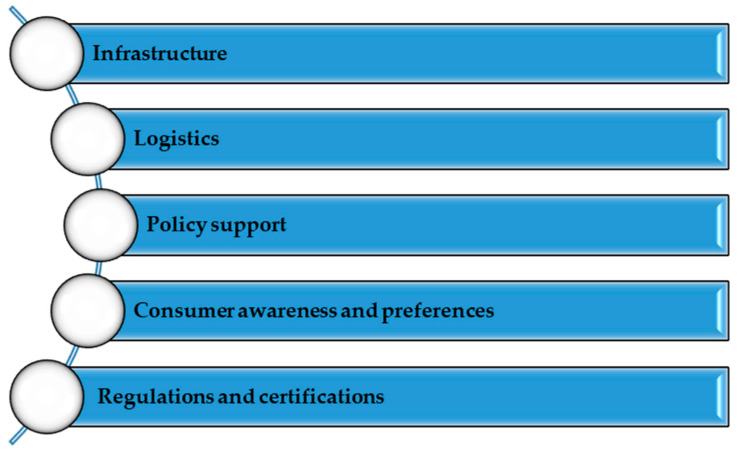
Challenges for short food supply chains. Source: Own study.

Developing short supply chains benefits consumers, producers, and the environment. However, some challenges may affect their effectiveness and development (Figure 2).

One of the main challenges for short supply chains is ensuring the proper infrastructure. For this chain type, farms and local producers must have access to adequate transport routes, cold stores, warehouses, and distribution points. The lack of sufficient infrastructure may cause supply difficulties, increase food losses, and limit the development of such chains. At the same time, it should be pointed out that the effective functioning of short supply chains requires efficient logistics. The optimization of supply routes, coordination between producers and consumers, and inventory management are crucial elements in ensuring the smoothness and efficiency of these types of chains. The lack of adequate logistics can lead to delays in deliveries, excessive costs, and difficulties in meeting consumer expectations. The development of short supply chains requires support from the authorities. Government policies, regulations, and programs to support local agriculture and small businesses can play a key role in promoting and facilitating the development of short supply chains. Policy initiatives may include financial incentives, administrative facilitation, or training programs for producers and suppliers to increase the efficiency and quality of these chains. Complex regulations and certifications often can be a barrier for small producers and suppliers wishing to participate in short supply chains. Sanitary and hygienic requirements, quality requirements, or ecological certifications can be time-consuming and costly for small enterprises. Simplifying procedures and lowering certification costs may encourage more producers to join such chains. The proper upbringing of consumers and education on the benefits of short supply chains are also crucial for their development. In many cases, consumers are not sufficiently aware of the advantages of such chains or do not prefer local and seasonal products. Promotion and education on short supply chains’ nutritional value and environmental benefits can change these preferences. In addition, short supply chains often compete with global supply chains, which have much more resources and can offer lower prices. This competition can be challenging for local producers, who may need help maintaining competitive prices and meeting market demands.

## 4. Results

### 4.1. Short Supply Chains and Food Policy for Healthy Eating

**Figure 3:**
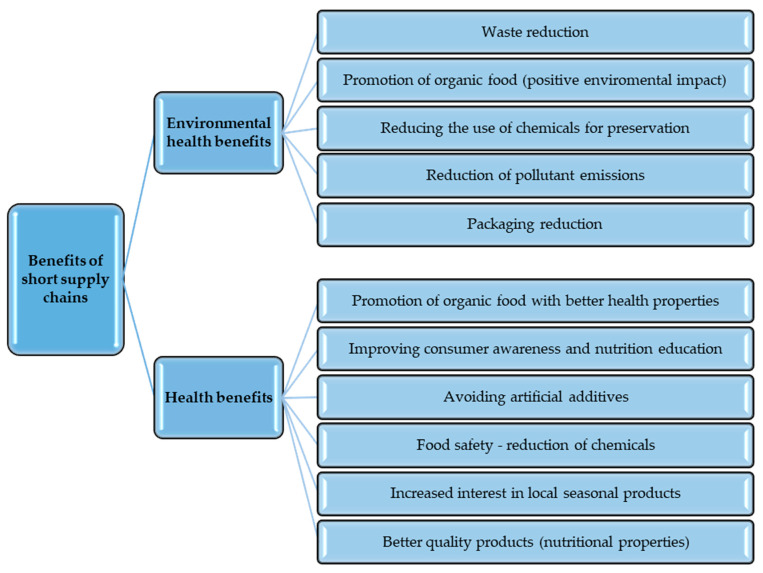
Benefits of short supply chains. Source: Own study.

It should be emphasized that the problem of healthy eating and access to high-quality food has become one of the main challenges for societies worldwide. We are becoming increasingly aware that our eating habits have a crucial impact on health and quality of life. However, pollution, long distances between producers and consumers, and dependence on supermarket chains make accessing fresh and healthy food more difficult. Hence, the already mentioned short supply chains answer these challenges. Their benefits are indicated in Figure 3.

Traditional, global food supply chains often require long transportation distances, which can lead to a loss of product quality. Fruits and vegetables must be harvested earlier to survive the journey, which can negatively impact their vitamin content and nutritional properties. In short food supply chains, food goes directly from the field to the plate without unnecessary intermediaries and long transportation routes, ensuring its quality and freshness. There is often a preference for local, seasonal products. This encourages sustainable and diverse food consumption, as consumers adjust their meals based on the ingredients available during a specific period. Seasonal fruits and vegetables are rich in vitamins and nutrients, contributing to a healthy diet. Increasing the availability of healthy and fresh products through short supply chains can also change the population’s eating habits. Consuming more natural and nutritious foods can lead to a reduced consumption of processed foods and thus may reduce exposure to unhealthy ingredients.

Short food supply chains also shorten the distance that food travels, reducing the risk of contamination and spoilage. Consumers can have greater confidence that their food is safe and comes from a reliable source. Additionally, shortening transportation reduces chemical usage, prolonging the freshness of fruits and vegetables. From a health perspective, it is also noteworthy that short food supply chains are less likely to contain artificial additives, preservatives, or artificial colors. Consumers have more control over the quality of products and can avoid potentially harmful substances.

It should be noted that short food supply chains also indirectly influence health through positive environmental benefits, which impact living conditions and public health. Short food supply chains often promote the production and distribution of organic food. Organic farms employ cultivation and breeding methods that minimize the negative impact on the environment. Synthetic pesticides, herbicides, and fertilizers are significantly reduced or eliminated, leading to fewer toxic substances in soil and water. This reduces exposure to harmful substances for farmers, consumers, and the ecosystems surrounding farms.

Short food supply chains allow also for a reduction in food waste. Traditional, global food supply chains often lead to overproduction, excessive packaging, and complicated distribution systems, contributing to the wastage of food products. In short food supply chains, food goes directly from the producer to the consumer, reducing losses and mitigating the negative environmental impact. A smaller quantity of food waste also means less burden on the natural environment, resulting in a healthier future for people and ecosystems. Moreover, these solutions enable avoiding excessive packaging. Products are often sold in reusable or even package-free containers, reducing the emissions of greenhouse gases associated with plastic production and limiting environmental pollution from microplastics.

Furthermore, it should be emphasized that short food supply chains, by connecting producers with consumers, encourage the use of natural methods for food preservation. Local storage or traditional processing methods often suffice, eliminating the need for the intensive use of chemical preservatives and pesticides. Avoiding the excessive use of chemical substances results in healthier food, free from toxic residues, positively impacting consumer health. Additionally, short food supply chains reduce the emissions associated with food transportation. The reduced impact on the natural environment promotes sustainable development and ecological conservation, affecting public health.

Overall, short food supply chains offer numerous environmental benefits that positively influence public health. Promoting organic food, reducing food waste, limiting chemical use, and avoiding artificial additives contribute to a healthier and more sustainable lifestyle for consumers. The combination of environmental advantages and health benefits makes short food supply chains vital in addressing global nutrition, food security, and environmental conservation challenges.

### 4.2. Forms of Short Supply Channels—Diagnosis in Poland

**Figure 4:**
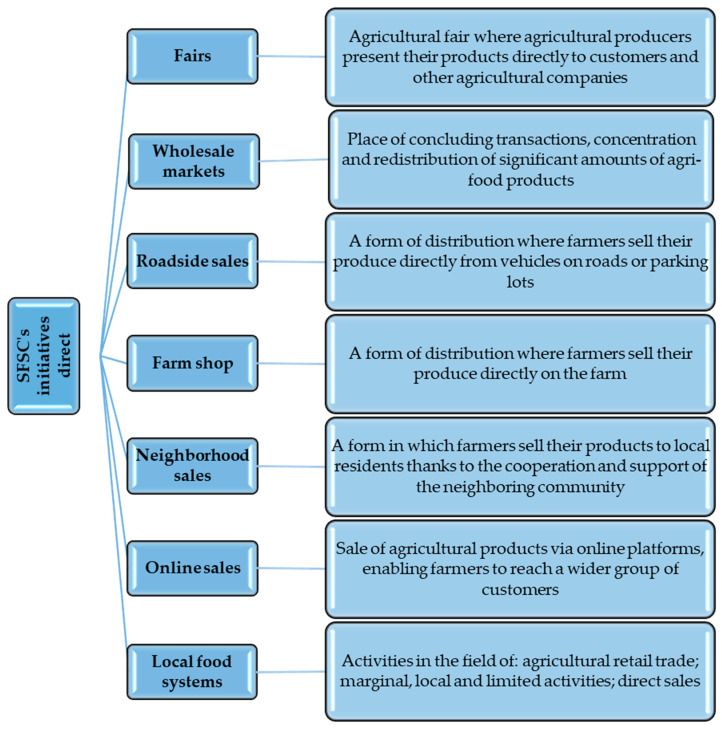
Forms of short direct channels. Source: Own study.

SFSC initiatives such as roadside sales and farmer’s shops have a low level of interdependence. On the other hand, socially supported agriculture requires direct and long-term interactions between producers and consumers. In the agri-food system, farmers, consumers, and public institutions pay special attention to fairness and transparency in setting prices and access to the market for small farmers, as well as counteracting the imperfections of competition. Short supply chains are expected to bring economic benefits to both producers and consumers. The former may obtain higher prices than sales to intermediaries (e.g., wholesale). Conversely, consumers may have access to cheaper and better-quality local food products. The main forms of short direct food supply chains in Poland are presented in Figure 4.

As already mentioned, the direct initiatives of the SFSC are characterized by a straightforward approach to the sale of agricultural products, enabling producers to establish closer contact with customers and other companies operating in the sector (Figure 3). These various sales methods offer innovative approaches to distribution, allowing to effectively reach local markets and meet the growing demand for fresh and locally sourced food products. In agricultural markets, producers can directly present their products to customers and enterprises related to the agri-food industry. It is a place where the exchange of information, knowledge, and experience plays a key role, and customers can establish direct contact with producers and learn more about production processes and the origin of products. On the other hand, wholesale markets are a platform for the concentration and redistribution of larger quantities of agri-food products. Here, transactions are made on a larger scale, which allows for products to be delivered to various destinations, including retail stores and restaurants. Forms of sale such as road sales and farm supply stores enable farmers to sell their products directly, eliminating intermediaries and allowing greater control over prices and quality. Roadside and farm sales are an innovative approach to reaching customers, which contributes to increasing the public awareness of local products. An important concept within the SFSC is also neighborhood selling, which is based on the cooperation and support of the local community. Here, farmers sell their produce to residents, creating a link between producers and consumers and strengthening the local economy. It should be noted that, in the digital transformation era, online sales are becoming an essential tool for farmers, allowing them to reach a broader range of customers through online platforms. This enables the sale of agricultural products on a larger scale while maintaining direct contact between the producer and the consumer. Many of the initiatives focus on creating local food systems, where agricultural retail, marginal activities, and direct sales play a crucial role. Striving to ensure access to fresh, locally sourced products becomes the foundation for a sustainable and socially responsible food future.

**Figure 5:**
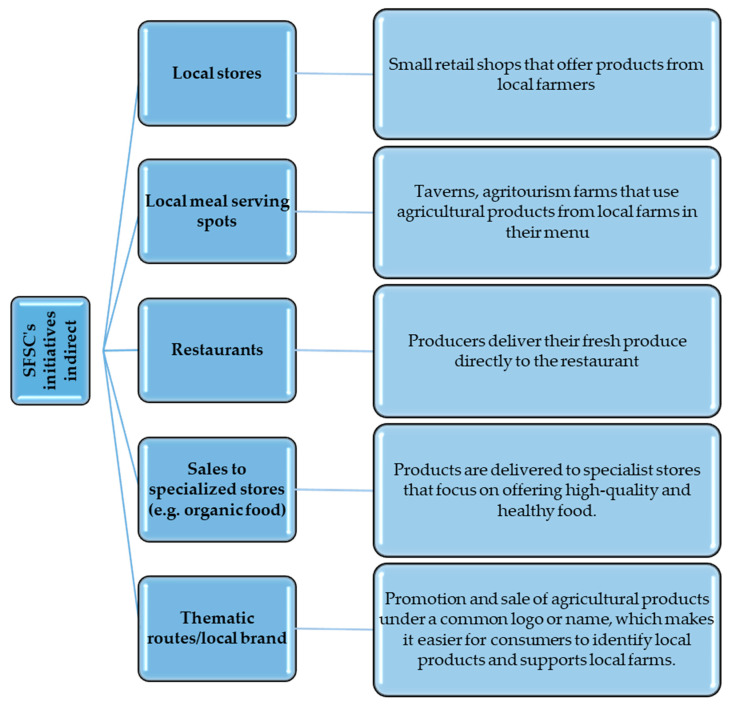
Forms of short indirect channels. Source: Own study.

Among the initiatives of short supply chains, one can also distinguish those operating in an indirect form (Figure 5).

Local stores are becoming a critical link in short indirect delivery channels, as they offer products straight from local producers. Customers can enjoy a selection of fresh and authentic produce while supporting farms. Local food outlets, such as inns and agritourism farms, use products from local farms in their menus. This enriches the culinary and tourist experience and creates synergistic links between the agricultural and tourism sectors. Restaurants also participate in short indirect delivery channels, accepting fresh commodities directly from producers. Thanks to this, customers are offered tasty, high-quality meals while supporting local farmers. Selling to specialized stores, such as health food stores or organic products, allows producers to reach more specialized markets. These stores emphasize high-quality and healthy products, which fits the SFSC philosophy. Thematic routes and local brands are initiatives promoting agricultural products under a common logo or name. This makes it easier for consumers to identify local products and increases their awareness of local farms. The concept of a theme route could include, for example, organized visits to farms, allowing consumers to learn about the production process and interact with farmers. In short, the SFSC’s short indirect supply channels highlight the close link between producers and consumers and promote local agricultural products as an essential element of a sustainable and socially responsible food chain.

### 4.3. Spatial Differentiation of Local Food Systems Registered in Poland

**Figure 6:**
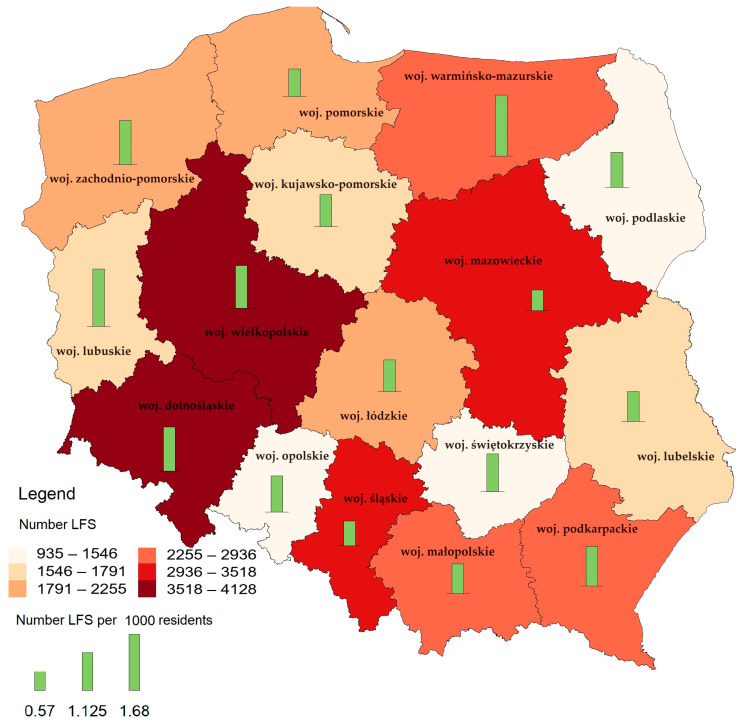
Number of entities operating under Local Food Systems registered in Poland (as of mid-2021) and number per 1000 residents. Source: Own elaboration based on data from the Chief Veterinary Inspectorate.

The total number of registered entities operating under Local Food Systems in Poland in mid-2021 was 35,178 (Figure 6). This overall represents the country’s commitment to developing local food networks. It should be noted that there are significant differences in the number of registered entities between regions. This may result from differences in local economic, agricultural, and cultural conditions. The leader in the number of entities registered as a part of the Local Food Systems is Wielkopolskie, in which 12% of the entities conducting these activities in Poland are registered. Dolnośląskie also has a significant number of registered entities, i.e., 10% of all entities. Mazowieckie, which is the capital region of Poland, also has a substantial number of entities registered as part of this activity (9%), which may result from the high diversity and availability of markets in this region. On the other hand, Opolskie has fewer registered entities than some other areas. This may suggest the need for greater involvement and the promotion of local products in this area. Thus, a comparison between regions reveals significant differences. Wielkopolskie and Dolnośląskie are characterized by a significant total number of entities operating under Local Food Systems, while Mazowieckie is a location for a significant number of these entities but with a lower number of them per capita (which may suggest significant potential for development in this area). Regional patterns in the location of Local Food Systems may result from the availability of markets, which is analyzed below.

**Figure 7:**
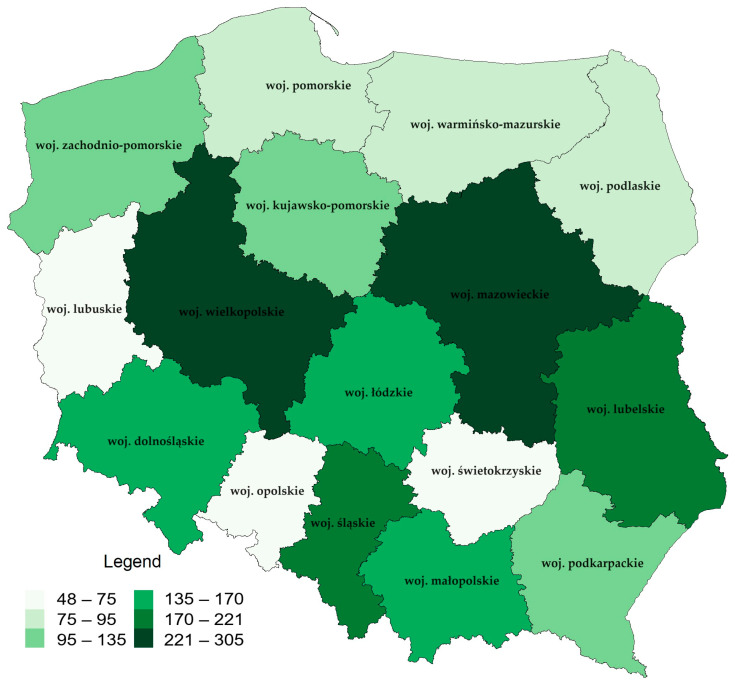
Number of permanent marketplaces in Poland at the end of 2022. Source: Own elaboration based on data from the Central Statistical Office (Local Data Bank).

As mentioned, marketplaces are important entities operating in short food supply channels. In 2021, there were a total of 2116 permanent marketplaces in Poland. This number reflects the presence of local commercial markets in different parts of the country. The regions with the highest number of marketplaces include Mazowieckie and Wielkopolskie. Mazowieckie has the most considerable number of permanent marketplaces (305 marketplaces) (Figure 7). This is understandable given the area’s economic and population importance. Wielkopolskie is another region with many permanent markets, which may result from this area’s commercial and agricultural traditions (221 markets). In the case of regions with the smallest number of marketplaces, Lubuskie should be indicated—73 marketplaces and the Opolskie—48 marketplaces. The distribution analysis shows that the numbers of permanent marketplaces in individual regions differs significantly. This distribution may be related to the economic, geographical, and cultural conditions of the regions. It should be noted that areas with fewer markets may require support and the development of commercial infrastructure. It is an essential tool for decision makers to shape the policy of supporting local trade and promoting the trade fair tradition in various regions of Poland.

**Figure 8:**
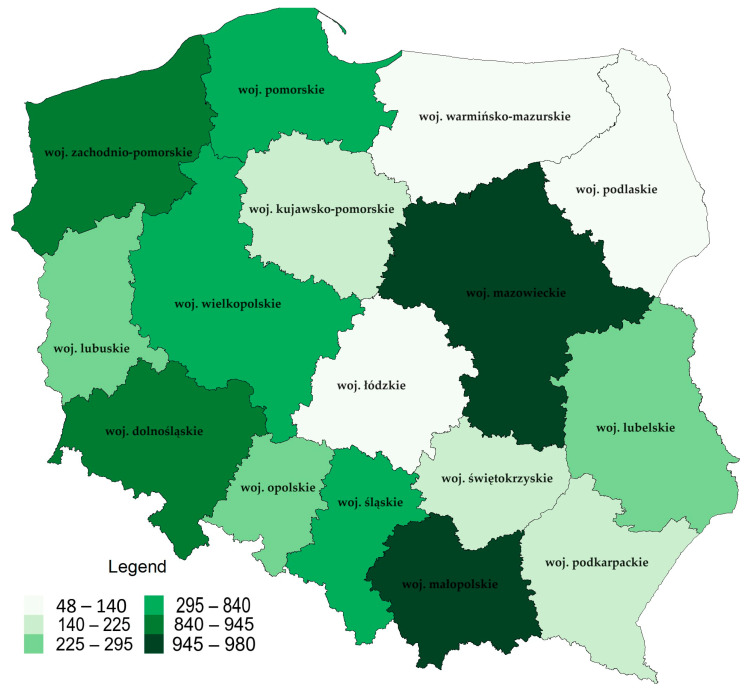
Number of seasonal markets in Poland at the end of 2022. Source: Own elaboration based on data from the Central Statistical Office (Local Data Bank).

In 2022, 6637 seasonal markets were present in Poland (Figure 8). This figure shows the sector’s massive impact on local economies and how it varies across the country regarding short supply channels. The statistics show that Mazowieckie is the area with the highest number of seasonal markets—980. Among the leaders in this respect, it is also worth mentioning Małopolskie—945 marketplaces, Zachodniopomorskie—932 marketplaces, Dolnośląskie—840 marketplaces, and Śląskie—810 marketplaces.

## 5. Discussion and Conclusions

Over the past few decades, short food supply chains and local food markets, where farmers sell their products directly to consumers or use a limited number of intermediaries, have developed worldwide in rural and urban areas. Some authors indicate even a kind of renaissance of traditional, direct ways of delivering food [27]. Short food supply chains complement conventional, often globalized chains, where small farmers have little bargaining power, and where disconnection between farmers and final consumers is observed [28]. It should be noted that, despite the trends mentioned above, determining the direction of the food system’s future development is very complex. An increasing number of consumers throughout the European Union seem to buy food products in shops at local marketplaces, directly on farms, or via basket/box delivery systems. European customers tend to associate local products with higher quality standards (freshness and nutritional value), healthy eating, more environmentally friendly production methods, and a lower carbon footprint. Moreover, the development of the concept of local food is implied by new trends in food consumption, referring to sustainable development in the environmental, social, and economic dimensions. The production and sale of local food positively affect not only the condition of the natural environment, but also foster the development of local communities, building relationships between producers and consumers and, consequently, improving quality of life. It is also pointed out to achieve local food self-sufficiency [29]. Interest in short food supply chains is also confirmed by the significant development of short food supply chain (SFSC) initiatives as alternatives to the globalized food chains typical of the modern food industry [30]. At the same time, it should be remembered that, in the case of supply chains, an important topic is the importance of a holistic view and the systemic nature of interactions between participants, which is both an advantage and an obstacle to more comprehensive implementation. On the one hand, the strategic nature of adopting a broad supply chain perspective provides significant potential benefits, while, on the other hand, it requires trading partners to think and act strategically [31], which is also relevant and applicable in the case of short food supply chains. At the same time, as suggested by [32], a holistic approach and insight into the issues/challenges of the SFSC can help to offer effective solutions and strategies for supporting the overall development of the SFSC. Bearing in mind the reference of this aspect to sustainable development, it should be noted that the resilience of agri-food systems around the world depends on the human ability to balance socio-economic and ecological trade-offs [33], and thus also the perception of these solutions, the potentials of sustainable development, and an important aspect in creating a healthy diet. Among other things, in this sector, it is necessary to consider sustainability and resilience criteria in evaluating and selecting food [34]. As the results of other researchers have indicated, sustainable development practices can positively increase the resilience of short food supply chains and vice versa [35]. In particular, social sustainability practices are seen as enablers of resilience, and production practices can have a positive or negative impact on resilience capacity [36]. In this context, it should be pointed out that the current food system, based on a long food supply chain (LFSC), is characterized by globalization. The literature emphasizes that LFSCs are unable to feed the world’s population and, moreover, generate negative ecological, environmental, logistic, and nutritional pressure. Therefore, innovative, efficient food systems that meet the current environmental and consumer requirements are required, as is the case with short food supply chains (SFSC) [37,38]. The state-of-the-art stresses that the importance of short supply channels and the issue of sustainable development of farms, due to their importance, are at the heart of the future interests of Agricultural Policy, not only in the economic and environmental aspects, but above all, within the limits of social analysis in connection with the newly introduced social interdependence [39,40,41,42].

All these factors are important from the perspective of LFS development in Poland. It should be noted that Poland is a significant food producer in Europe. Hence, it seems vital to strive for the development of Local Food Systems. It should be noted that substantial spatial differences characterize the activities of Local Food Systems in Poland. LFS entities are also differentiated due to the legal form of their organization, which may result in a different business model [43].

It should be noted that the available datasets do not cover the sale of products of plant origin, i.e., fruit and vegetables, under Local Food Systems. It is all the more important that the fruit and vegetable market is distinguished by autonomous local markets, a high seasonality, a significant share of small, unorganized producers, and a wide range of products that differ in quality [44]. In addition, due to the great importance of this production in the study area, especially in the central and southern parts of Poland, it is essential to take action to fill this information gap as part of future research.

In conclusion, short supply chains are gaining more and more popularity due to their potential for improving food quality and consumer health. An analysis of the available literature indicates that these benefits are due to several key factors. The first of these factors is the direct communication between the producer and the consumer, which is characteristic of short supply chains. Research results confirm that this direct interaction increases transparency and control over food quality. Food from short supply chains appears to be more concentrated regarding nutritional value, translating into healthier eating for consumers. Short supply chains also positively impact preserving the freshness and flavor of food. Fast transport from the producer to the consumer minimizes storage time, translating into a longer product shelf life. The next important aspect of short supply chains is their focus on local and seasonal products. This approach contributes to a balanced and healthy diet by allowing access to fresh fruit, vegetables, meat, and dairy products available in season. Consumer education also plays a vital role in short supply chains, informing people about healthy eating, the nutritional value of products, and the role of local and organic practices. Furthermore, the role of short supply chains in supporting local economies and small producers must be remembered. Practical examples show that these chains contribute to the development of local communities and promote sustainable production. The conclusions from the literature analysis are clear: short supply chains bring about numerous benefits in terms of food quality, consumer health, and supporting local economies. In the context of the global need to promote healthy eating and sustainable consumption, short supply chains are a valuable alternative that deserves further research and implementation.

Although consumers and producers increasingly prefer more direct and transparent food distribution channels, such as short food supply chains (SFSC), they face various problems and challenges in their development and operation, resulting in a limited efficiency and sustainability, as well as difficulties in scaling up. It should be noted that the conducted research has limitations, and further research is recommended to supplement the knowledge on this topic, mainly in terms of extending the time scale by continuing observations, increasing the consumer’s approach to using direct sales, and also e-manufacturers regarding cooperation in these distribution chains. Other limitations refer to regional and national contexts, which do not allow for the generalization of the results to a global level. Moreover, the variability in time factors may affect the dynamic nature of short supply chains. The lack of clear health indicators and definitions of short supply chains may introduce uncertainty in the interpretation of data.

Nevertheless, this study makes an important contribution to the field of research on short supply chains, sustainable development, and healthy eating, especially in the context of Poland. By applying various research methods and analyzing a wide range of sources, the paper helps to understand the role of short supply chains in the context of food policy and healthy eating. Through the analysis of the practical aspects of short supply chains, i.e., field observations, the study allows for a better understanding of the functioning of these chains, their role in supporting local farmers, and their impact on healthy nutrition the society.

The paper provides practical implications for the policymakers and practitioners involved in developing sustainable food strategies. By analyzing the economic benefits for local producers, the impact on consumer health, and innovation practices in Poland, the article can serve as a guide for decision makers who want to promote sustainable development in the food sector.

## Data Availability

Publicly available datasets on the number of markets were analyzed in this study. This data can be found here: [https://bdl.stat.gov.pl/bdl/dane/podgrup/temat, accessed on 11 November 2023]. The data presented in this study on the number of entities operating under Local Food Systems registered in Poland are available on request from the Chief Veterinary Inspectorate. The data are not publicly available due to legal regulations on the organisation of this institution.
